# Next-generation reopenable-clip over-the-line method for gastric defects after endoscopic submucosal dissection at an anastomotic site

**DOI:** 10.1055/a-2853-6021

**Published:** 2026-05-06

**Authors:** Tatsuma Nomura, Fumiya Hasegawa, Ken Ichikawa, Morihito Setsuda, Takashi Hamada, Kaneko Hiroshi, Katsumi Mukai

**Affiliations:** 1 Department of Gastroenterology 36951 Suzuka General HospitalSuzukaJapan; 2 Department of Endoscopy Center 36951 Suzuka General HospitalSuzukaJapan; 3 Department of Surgery 36951 Suzuka General HospitalSuzukaJapan

**Fig. 1:**
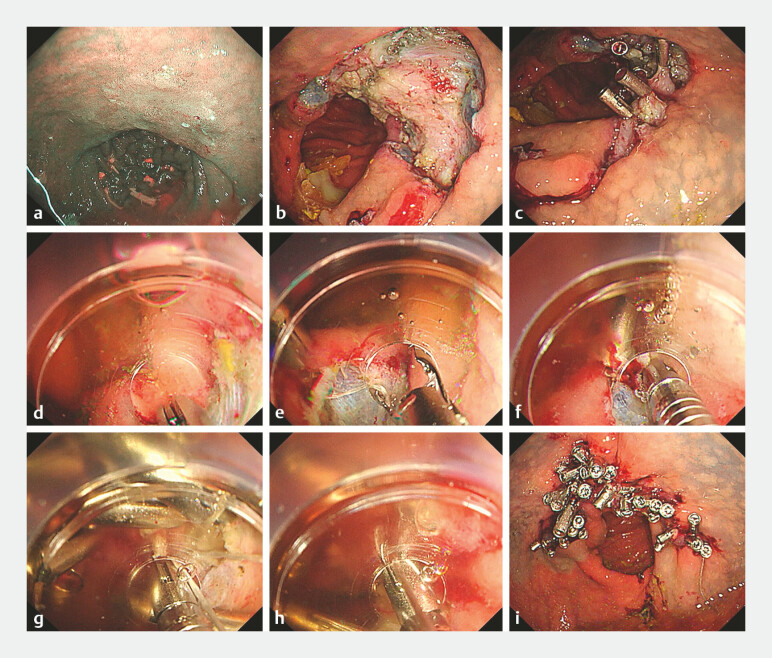
Closure of a post-ESD gastric defect at an anastomotic site using the next-generation ROLM. **a** Early gastric cancer measuring approximately 30 mm located on the anastomotic line. **b** A post-ESD gastric defect measuring approximately 50 mm. **c** A reopenable clip with a line equipped with small anti-slip protrusions is placed to simultaneously grasp the mucosal and muscular layers at one edge of the defect. **d** A reopenable clip with a line threaded through a hole in one of its teeth is applied to grasp the mucosal and muscular layers at the contralateral edge of the defect. **e** and **f** The line passes through the hole in the clip tooth on one side of the defect. **g** and **h** Traction is applied to the line from the endoscope while maintaining firm grasp of the mucosal and muscular layers, approximating the defect edges. Sequential clip placement stabilizes the defect edges through friction generated among the clip tooth hole, line, and grasped tissue. **i** Complete closure of the post-ESD gastric defect achieved using next-generation ROLM. ESD, endoscopic submucosal dissection; ROLM, reopenable-clip over-the-line method.

Various endoscopic techniques have been developed for defecting closure following gastric endoscopic submucosal dissection (ESD) for early gastric cancer 1 . We previously proposed the reopenable-clip over-the-line method (ROLM), which achieves defect closure by utilizing friction generated between a line (PE line: Super X-wire 1.5, DUEL Co.), the teeth of reopenable clips, and grasped tissue 2 3 . Recently, a novel reopenable clip equipped with small anti-slip protrusions (Lockado clip, 11 mm; Micro-Tech Co. Ltd, Nanjing, China) has been introduced to enable stronger tissue fixation 4 . Here, we report a case in which next-generation ROLM using a Lockado clip was successfully applied for closure of a post-ESD gastric defect at an anastomotic site, which is considered technically challenging. A man in his 70s was diagnosed with early gastric cancer located on the anastomotic line at the lesser curvature of the upper gastric body after distal gastrectomy. We performed en bloc resection using ESD ( Fig. 1 and Video 1 ). The resulting mucosal defect measured approximately 50 mm and extended along the entire anastomotic line, making short-axis closure difficult. Therefore, double-layered suturing using the reopenable-clip over-the-line method was initially performed to reduce the defect along the short axis 5 . The defect was then closed using next-generation ROLM. First, a clip with a line was placed on the anal side of the defect to grasp both the mucosal and muscular layers. Additional reopenable clips with a line threaded through one tooth were sequentially applied to grasp the mucosal and muscular layers at the edges of the defect. By pulling the line while maintaining firm tissue grasp, the defect edges were approximated and strongly fixed through enhanced friction between the line, the small clip holes, and the anti-slip protrusions. Repetition of this maneuver enabled the complete closure of the defect on the anastomotic site without burying the muscle-grasping clips. The patient was discharged without adverse events.

Closure of a gastric defect at an anastomotic site using a reopenable clip with small anti-slip protrusions (next-generation ROLM). ROLM, reopenable-clip over-the-line method.Video 1

Endoscopy_UCTN_Code_TTT_1AO_2AO
